# Nanobody Mediated Crystallization of an Archeal Mechanosensitive Channel

**DOI:** 10.1371/journal.pone.0077984

**Published:** 2013-10-21

**Authors:** Christian Löw, Yin Hoe Yau, Els Pardon, Caroline Jegerschöld, Lisa Wåhlin, Esben M. Quistgaard, Per Moberg, Susana Geifman-Shochat, Jan Steyaert, Pär Nordlund

**Affiliations:** 1 Department of Medical Biochemistry and Biophysics, Karolinska Institutet, Stockholm, Sweden; 2 Division of Chemical Biology and Biotechnology, Nanyang Technological University, Singapore, Singapore; 3 Structural Biology Research Center, VIB, Vrije Universiteit Brussel, Brussels, Belgium; 4 Structural Biology Brussels, Vrije Universiteit Brussel, Brussels, Belgium; 5 Department of Biosciences and Nutrition, Karolinska Institutet and School of Technology and Health, Royal Institute of Technology, Huddinge, Sweden; 6 School of Biological Sciences, Nanyang Technological University, Singapore, Singapore; University of Houston, United States of America

## Abstract

Mechanosensitive channels (MS) are integral membrane proteins and allow bacteria to survive sudden changes in external osmolarity due to transient opening of their pores. The efflux of cytoplasmic osmolytes reduces the membrane tension and prevents membrane rupture. Therefore these channels serve as emergency valves when experiencing significant environmental stress. The preparation of high quality crystals of integral membrane proteins is a major bottleneck for structure determination by X-ray crystallography. Crystallization chaperones based on various protein scaffolds have emerged as promising tool to increase the crystallization probability of a selected target protein. So far archeal mechanosensitive channels of small conductance have resisted crystallization in our hands. To structurally analyse these channels, we selected nanobodies against an archeal MS channel after immunization of a llama with recombinant expressed, detergent solubilized and purified protein. Here we present the characterization of 23 different binders regarding their interaction with the channel protein using analytical gel filtration, western blotting and surface plasmon resonance. Selected nanobodies bound the target with affinities in the pico- to nanomolar range and some binders had a profound effect on the crystallization of the MS channel. Together with previous data we show that nanobodies are a versatile and valuable tool in structural biology by widening the crystallization space for highly challenging proteins, protein complexes and integral membrane proteins.

## Introduction

In spite of recent developments of improved protein expression and purification tools for integral membrane proteins (IMPs), the preparation of diffraction quality crystals remains the major bottleneck for their structure determination by X-ray crystallography [1]. Two major reasons for this are the conformational heterogeneity of many IMPs in solution, and the presence of detergents, which limit the surface area available for forming ordered crystals of IMPs. IMPs, such as G-protein coupled receptors, channels and transporters function through conformational changes and therefore exist in an ensemble of functionally distinct states [2-7]. Extraction of these proteins from the natural membrane environment in a detergent solution might enhance conformational dynamics contributing to increased sample heterogeneity and lower success rates in crystallization. A promising approach to increase the likelihood of crystal formation and to improve diffraction quality is the use of crystallization chaperones [8-11]. These chaperones typically represent macromolecules that have been selected to bind specifically to a given target protein. Ideally, they i) bind to a specific conformation reducing conformational heterogeneity and ii) provide additional protein surface for productive crystal contact formation. Furthermore, crystallization chaperones can provide initial model-based phasing information. Fragments of monoclonal antibodies represent classical crystallization chaperones, but this traditional approach is time consuming and expensive. Small crystallizable proteins from combinatorial libraries have also been developed to further expand the crystallization toolbox [12-18]. In addition to classical Fab´s, camelid VHH domains (variable heavy chain domain of a camelid heavy chain only antibody), also called nanobodies, derived from immunized llamas have gained attention due to their versatility of binding modes [19-27]. Indeed, the presence of a nanobody has been critical for the structure determination of a number of soluble proteins and the recently described IMP structures of the activated β_2_AR and β_2_AR-G-protein complex (beta-2-adrenergic receptor GTP binding protein) [28,29].

Mechanosensitive (MS) channels were identified as emergency valves when bacteria experience significant environmental stress. The transient opening of their pores upon sudden changes in external osmolarity (osmotic shock) leads to efflux of cytoplasmic osmolytes, which reduces the membrane tension and prevents membrane rupture. Therefore MS channels allow the organism to survive and grow in a wide range of external osmolarities [30-35]. Two families of MS channels have been identified in bacteria: The MS channels of large and small conductance (MscL and MscS, respectively), [34-36]. Members of both families are widely distributed in all kingdoms of life, and many organisms express multiple family members [31,37]. The MscS family is one of the best characterized MS channel family and crystal structures are known of MscSs of *Escherichia coli* [38-40], *Helicobacter pylori* [40] and *Thermoanaerobacter tengcongensis* [41]. Since members of the MscS family show great variability in size and sequence, we have in this study focussed on the structural characterization of two different MscS members from the archaea *Thermoplasma volcanium*. Members of the *Thermoplasma* genus lack cell walls. Instead, they are surrounded by a membrane, mainly composed of tetraether lipoglycans, which display high resistance towards acid and heat, thus enabling these organisms to live under extreme conditions [42]. 

Here we report the high-level expression, purification and biophysical characterization of two MS channels from *Thermoplasma volcanium* (T1 and T2) and the characterization of nanobodies obtained from immunized llamas against the T2 channel. T2 specific nanobodies bound with high affinity (*K*_D_ in the pM to nM range) and had a profound effect on its crystallization.

## Materials and Methods

### Ethical statement

All animal vaccination experiments were executed in strict accordance with good animal practices, following the EU animal welfare legislation and after approval of the local ethical committee (Committee for the Use of Laborary Animals at the Vrije Universiteit Brussel, VUB). VUB specifically approved the full study. Every effort was made to minimize suffering.

### Materials and reagents

All detergents were purchased from Affymetrix. Luria-Bertani (LB) and Miller and Terrific broth (TB) were from Formedium. Kanamycin and carbenicillin were obtained from Duchefa Biochemie and Isopropyl β-D-1-thiogalactopyranoside (IPTG) was from Saveen Werner. Chemicals and consumables for surface plasmon resonance (SPR) analysis were purchased from GE Healthcare. All other chemicals were from Sigma-Aldrich, unless otherwise stated. 

### Construct design of IMPs

The genes coding for the two mechanosensitive channels from *Thermoplasma volcanium* (named T1 (accession no. TVN0705) and T2 (accession no. TVN0821)) were amplified from genomic DNA (DSM 4299 [GSS1*]) and cloned into the N-terminal His-tag vectors pNIC28-Bsa4 and pET46 using ligation independent cloning [43,44]. Both vectors possess a cleavage site for tag removal: Tobacco Etch Virus (TEV) cleavage site in the pNIC28-Bsa4 vector and Enterokinase cleavage site in the pET46 vector. All vectors possess a T7 promoter and terminator sequence. The correct insertion of the gene sequence was verified by DNA sequencing.

### Small scale protein expression and membrane preparation

Both channels were over-expressed in *E. coli* BL21(DE3), C41(DE3), C43(DE3) and Rosetta 2(DE3) cells and analyzed as previously described [45]. Cultures of 100 ml TB medium in 300 ml baffled conical flasks were inoculated from a LB overnight culture to a start optical density at 600 nm (OD_600nm_) of 0.05 and grown at 37 °C at 200 rpm. At an OD_600nm_ of 0.7 - 1.0, the cultures were induced with 0.2 mM IPTG for either 16 hours at 20 °C, 8 hours at 30 °C or 4 hours at 37 °C prior to harvest. Cell density was monitored by measuring the OD_600nm_ value. 90 ml of the cultures were harvested at 5,000 × g for six minutes and the cell pellets were stored at -80 °C.

Frozen cell pellets were thawed on ice and resuspended in 5 ml lysis buffer per g of cells (wet weight) (20 mM Tris pH 8.0, 300 mM NaCl, 1 mg/ml lysozyme, 5 U/ml DNase I, 100 x diluted EDTA free complete protease inhibitor cocktail (Roche)) and lysed by using a high pressure homogenizer (Avestin). Crude membranes were harvested using ultracentrifugation at 104,000 × g (Beckman Coulter Ti45 rotor) for 50 min. Thereafter, membranes were resuspended in solubilization buffer (20 mM sodium phosphate buffer pH 7.5, 300 mM NaCl, 20 mM imidazole, 0.5 mM TCEP, 5 % glycerol and 100 × diluted EDTA free protease inhibitor cocktail (Roche); 3 ml of solubilization buffer per 200 OD_600nm_ units). Aliquots were flash-frozen in liquid nitrogen and stored at -80 °C until further use. 

### Protein over expression and purification – T1 and T2 channel

Both mechanosensitive channels, T1 and T2, were over-expressed in *E. coli* C41(DE3) and C43(DE3) cells, respectively. Cultures of 1 L TB medium in 2.5 L baffled conical flasks were inoculated from a LB overnight culture to a start OD_600nm_ of 0.05 and grown at 37 °C at 200 rpm. At an OD_600nm_ of 0.7 - 1.0, the temperature was reduced to 20 °C over 60 min followed by IPTG induction (0.2 mM). Cultures continued to grow for additional 16 hours prior to harvest. Cell density was monitored by measuring the OD_600nm_ value. Cells were harvested at 10,000 × g for 10 minutes and cell pellets were stored frozen at - 80 °C. For cell lysis, 5 ml of lysis buffer (20 mM sodium phosphate buffer pH 7.5, 300 mM NaCl, 5 % glycerol, 1 mg/ml lysozyme, 5 U/ml DNase I, 100 x diluted EDTA free complete protease inhibitor cocktail (Roche)) was added per gram of cells (wet weight). The resuspended cells were then incubated under stirring at 4 °C for 45 min and disrupted with an Emulsiflex microfluidizer (Avestin) at 15,000 p.s.i. chamber pressure. Unbroken cells and cell debris were removed by centrifugation at 10,000 × g for 10 min at 4 °C and the membranes were collected by ultracentrifugation at 104,000 × g (Beckman Coulter Ti45 rotor) at 4 °C for 50 min. Membranes were resuspended in solubilization buffer (3 ml buffer/200 OD_600nm_ units; 20 mM sodium phosphate buffer pH 7.5, 300 mM NaCl, 15 mM imidazole, 0.5 mM TCEP and 5 % glycerol, 100 × diluted EDTA free complete protease inhibitor cocktail (Roche)) supplemented with 1 % DDM (dodecyl-β-D-maltoside). After 60 min of stirring at 4 °C, solubilized membranes were centrifuged to remove unsolubilized material (104,000 × g at 4 °C for 30 min). 

Preparative immobilized metal affinity chromatography (IMAC) was performed by batch-adsorption of 140 - 200 ml of solubilized membranes by end-over-end rotation with 6 ml (settled) Ni Sepharose^TM^ 6 Fast Flow resin (Invitrogen) for 45 min. The resin was then packed in a 10-mm-(i.d.) open gravity flow column and unbound proteins were removed by first washing with 10 bed volumes of solubilization buffer supplemented with 0.03 % DDM (buffer content; see above) and then washing with ten bed volumes of solubilization buffer containing 30 mM imidazole. The proteins were then eluted by addition of five bed volumes of solubilization buffer containing 500 mM imidazole. Directly after elution, the target protein was transferred to a dialysis bag (cut-off 10 kDa) and either recombinant TEV protease or Enterokinase was added to a final concentration of 0.5 - 2 µM. Dialysis against imidazole free solubilization buffer was performed overnight at room temperature. All buffers contained 0.03 % DDM. After tag cleavage, both channel proteins were subjected to another IMAC purification step. Here, the flow through fraction with tag free and highly purified protein was collected and concentrated to 5 ml using a 100 kDa cut-off concentrator (Sartorius Stedim Biotech VIVASPIN 20). Thereafter the concentrated protein was loaded on a HiLoad Superdex^TM^ 200 16/60 GL column using an ÄKTAexplorer^TM^ 10 chromatography system. Peak fractions were pooled and concentrated to 15 - 30 mg/ml protein. The protein samples were either flash frozen in liquid nitrogen and stored at - 80 °C, or preferably used directly for crystallization trials. For the preparation of various T2/nanobody complexes, the T2 channel was incubated with an excess of nanobody and further purified on preparative gel filtration in 20 mM sodium phosphate pH 7.5, 150 mM NaCl, 5 % glycerol, 0.03 % DDM. Peak fractions containing the channel protein and the nanobody in stoichiometric amount were used for subsequent crystallization trials.

### Analytical gel filtration

To assess the quality of the purified membrane protein, IMAC eluted samples were analyzed by gel filtration on a Superdex^TM^ 200 5/150 GL size exclusion column using an ÄKTAmicro^TM^ chromatography system (GE Healthcare) equipped with the autosampler A-905, which automatically injected 25 µl of protein sample. Analytical gel filtration runs (AGF) were performed at 4 °C at a flow rate of 0.2 ml/min in gel filtration buffer (20 mM sodium phosphate pH 7.5, 150 mM NaCl, 5 % glycerol, 0.03 % DDM).

To monitor complex formation, the T2 channel (0.3 mg/ml) was incubated at least with a two fold excess of nanobody for 1 hour and subsequently analysed in duplicates on the described AGF set-up. As control, both proteins were also run separately on the AGF column. To demonstrate specificity of binding, all selected nanobodies against the T2 channel were also analyzed for complex formation with the T1 channel on AGF.

### SDS-PAGE and Western Blots

For gel electrophoresis, NuPAGE 4-12% Bis-Tris Gels (Life Technologies^TM^) were used and stained by Coomassie Brilliant Blue R-250. Mark12 standard (Life Technologies^TM^) or SeeBlue® Plus2 Prestained (Life Technologies^TM^) were used as protein markers for SDS-PAGE and Western blots (WB), respectively. For Western blotting, proteins were transferred to nitrocellulose membranes using an iBLOT^TM^ blotting system (Life Technologies^TM^). Blots were blocked using 1 % bovine serum albumin (BSA) in TBS-T buffer (20 mM Tris (pH 7.5), 100 mM NaCl, 0.05 % (v/v) Tween® 20) for 1 hour at room temperature. Membranes were washed 3 times for 10 minutes with TBS-T buffer and incubated with the different nanobodies at a concentration of 1 µg/ml for 1 hour. After another wash step (3 times 10 minutes with TBS-T buffer) the membrane was incubated with a horseradish peroxidase-labelled His-probe (HisProbe^TM^-HRP, Thermo Scientific) that recognizes poly-histidine tagged fusion proteins. Western blots were developed with Super Signal West Pico (Thermo Scientific) chemiluminescent substrate. Signals were detected and quantified using a Fluor-S^TM^ MultiImager (Bio-Rad). Reported intensity values represent the mean values with standard deviations of three independent experiments (N=3).

### Generation and purification of T2 specific nanobodies

A llama was immunized six times with 330 μg of purified recombinant T2 channel protein in detergent solution over a period of 6 weeks. From the anti-coagulated blood of the immunized llama, lymphocytes were used to prepare cDNA which served as a template to amplify the open reading frames coding for the variable domains of the heavy-chain antibodies. The PCR fragments were ligated into the pMESy 2 phagemid vector, a derivative of pMES4 (genbank GQ907248) carrying the CaptureSelect^TM^ C-tag (i.e. C-terminal EPEA) [24] instead of the His-tag and transformed in *E. coli* TG1 cells. The VHH repertoire of this library was expressed in phages after super infection with helper phages, and selection of phage particles expressing nanobodies that bind the T2 channel was performed. Phages were recovered by incubating the T2-channel-coated wells with 100 mM triethylamine (pH 10) for 10 min. These T2-channel-coated wells were then washed once with 50 mM Tris-HCl (pH 6.8) and several times with PBS, and freshly grown TG1 cells were added to the wells to recover the non eluted phages. A clear enrichment was observed after two consecutive rounds of selection on solid-phase-coated antigen. Twice, 72 randomly chosen colonies — after the first and second round — were grown for expression of their nanobody as soluble protein. Crude periplasmic extracts were tested in an ELISA, and 102 extracts were shown to be specific towards the T2 channel. From the positive clones, the VHH genes were amplified by PCR and a HinfI restriction fragment length polymorphism was performed on all of them. Sequence analysis on 47 clones revealed 33 different nanobodies against the T2 channel. Finally, 23 selected nanobody genes were cloned in a pHEN6 vector for recombinant expression with a His-tag in the *E. coli* (SI Figure 1: Figure Sequence alignment). Nanobodies bearing a C-terminal His-tag were expressed in the periplasm of *E. coli* strain WK6 following induction with IPTG. Cultures of 0.5 L were grown to OD_600_ = 0.7 at 37 °C in TB media containing 0.1% glucose, 2 mM MgCl_2_, and 50 μg/ml carbenicillin, induced with 1 mM IPTG and grown overnight at 28 °C. Cells were harvested by centrifugation and the periplasmic fraction was prepared *via* osmotic shock. Therefore cells were resuspended in ice-cold buffer (50 mM Tris pH 8.0, 12.5 mM EDTA, and 0.125 M sucrose) and cell debris were removed by centrifugation. Nanobodies were purified from the periplasmic extract by immobilized-metal affinity chromatography followed by gel filtration (20 mM Tris pH 7.5, 150 mM NaCl), and concentrated to 10 - 20 mg/ml [46].

**Figure 1:**
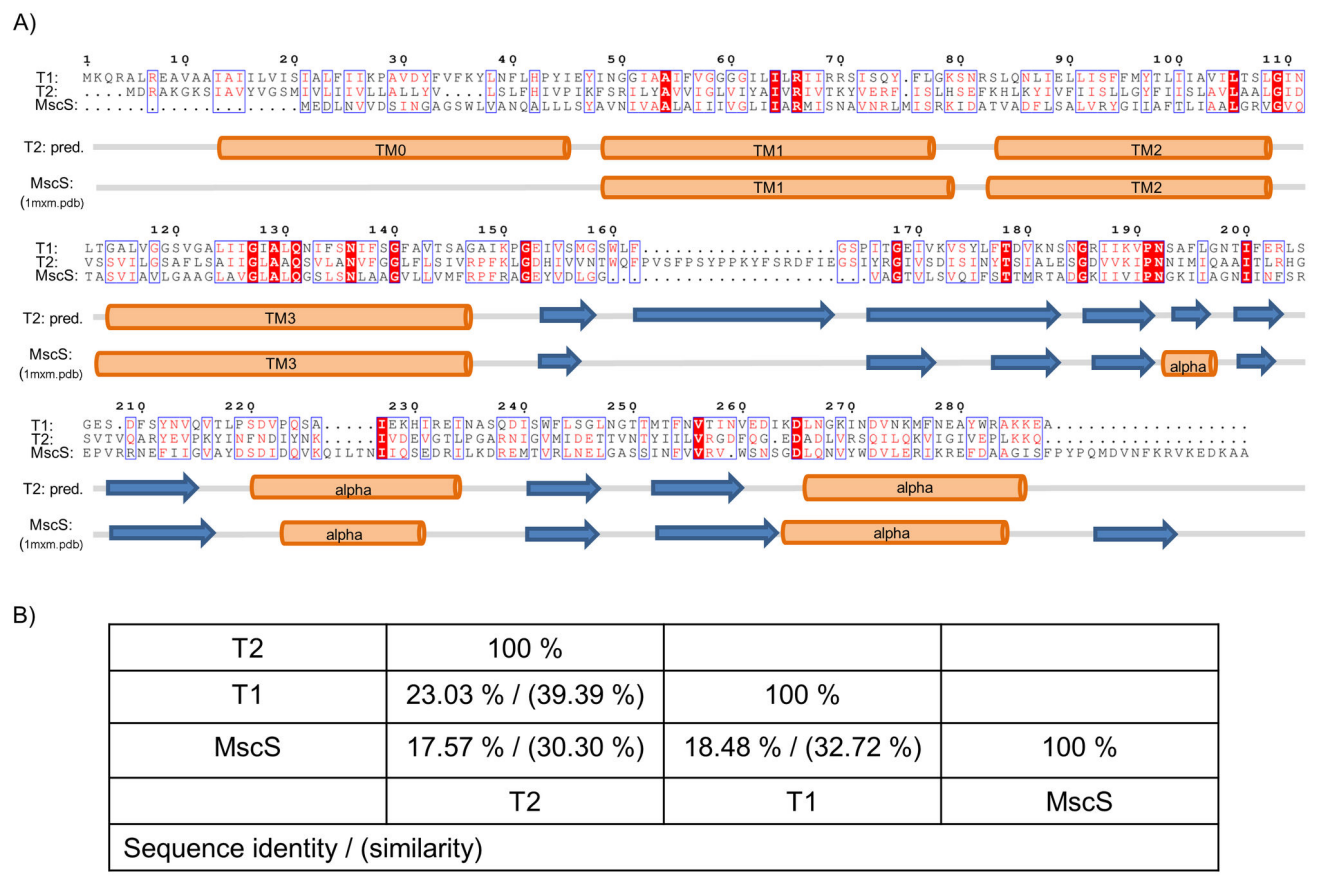
Sequence alignment and secondary structure of bacterial and archeal mechanosensitive channels. (A) Sequence comparison of the archeal mechanosensitive channels T1 and T2 from *Thermoplasma volcanium* with MscS from *E. coli*. Identical and similar residues are boxed and color coded in red. Highest sequence similarity is found in transmembrane helix 3. Secondary structure elements based on the bioinformatic prediction tool Jpred 3 for T2 [65] and on the crystal structure of MscS (1mxm.pdb) is shown underneath the alignment. (B) Sequence identities and similarities between T1, T2 and MscS as calculated using the pairwise identity server SIAS [66].

### Surface plasmon resonance analysis

All measurements were performed on a BIAcore^®^ 3000 instrument at 25 °C using 20 mM sodium phosphate buffer, pH 7.4 containing 150 mM NaCl and 0.03 % DDM. Buffer was degassed and filtered through a 0.2 μm filter. 

Immobilizations were performed at a flow rate of 10 μl/min. T2 channel protein was covalently immobilized on the surface dextran of a sensor chip CM5 through amine-coupling. The carboxyl group on the dextran matrix in a flow channel was activated with a 7-minute injection of a 1:1 mixture of 0.1 mM N-hydroxysuccinimide (NHS) and 80 mM 1-ethyl-3-dimethylaminopropyl-carbodiimide (EDC). T2 channel protein (19.7 μM) was diluted into 10 mM sodium acetate buffer pH 5.0 and the diluted protein was injected across the activated surface until an immobilization level of 1500 - 2000 RU was reached. The surface was then inactivated with a 7-minute injection of 1 M ethanolamine-HCl. An unmodified flow channel was used as a reference surface for all experiments. Kinetic measurements for all nanobodies were performed at a flow rate of 30 μl/min. Three-fold serially-diluted nanobodies at 0.35, 1.1, 3.2, 9.6, 28.7 and 86.1 nM were injected in triplicates for 2 min followed by another 30 min of dissociation in running buffer. Surface was regenerated with a quick pulse (30 sec) of 20 mM HCl at the end of each cycle. 

All raw data collected were processed using Scrubber2 software (BioLogic Software Pty Ltd, Australia). Raw sensorgrams were first corrected with data from the reference channel and subsequently corrected with blank buffer cycles. A set of corrected sensorgrams of a concentration series for a nanobody was globally fit to a simple bimolecular interaction model to obtain its rate of association (*k*_a_) and rate of dissociation (*k*_d_). The affinity constant, *K*_D_, was calculated from the ratio of *k*_d_/*k*_a_. Replicates (n = 3) of these values were used to calculate the experimental standard errors. Final figures were prepared using GraFit 5.

### Electron microscopy

The channels were diluted to 0.011 mg/ml before the adsorption onto glow discharged carbon coated copper grids (400 mesh) for 1 minute. Grids were washed with 3 drops of double distilled water and stained with one drop of 1% uranyl formate. Images were acquired on a JEOL2100F electron microscope using a 4K × 4K CCD camera (Tiez Video and Imaging Processing System GmbH, Germany). The images were taken at an underfocus around 2.3 mm at an accelerating voltage of 200kV and magnification of 60 000. The pixel size of the CCD camera was 15 mm (corresponding to 1.8 Å at the specimen level).

### Protein crystallization

Before crystallization setup, the IMP/nanobody samples were centrifuged at 20,400 × g in a cooled bench top centrifuge for 10 min. Crystallization trials were performed using a mosquito (TTP LabTech) crystallization robot with a total drop volume of 300 nl (at 3 different protein/precipitant ratios: 1:1, 1:2 and 2:1). The two sparse matrix screens, ProComplex and JCSG+ (Qiagen) were used for initial crystallization trials at 20 °C. Crystal growth was followed over 21 days using the Rock Imager software (Formulatrix). Crystals were flash frozen and analyzed for diffraction at the synchrotron. For complex verification, crystals were collected, dissolved in SDS loading buffer and analyzed on SDS PAGE.

## Results

### Sequence analysis of the T1 and T2 channels

The MS channels of small conductance (MscS) from the Archaea Thermoplasma *volcanium* (T1 and T2) were identified as prime candidates for structural studies using our recently established screening pipeline [47].They are well expressed and yield a monodisperse gel filtration peak in various detergents. Despite limited sequence conservation (see Figure 1), both channels are annotated as members of the MscS family, which is much larger and more variable in size and sequence than the MS channels of large conductance (MscL). Overall, the secondary structure predictions of T1 (288 amino acids per monomer, M_w_ =31.5 kDa) and T2 (297 amino acids per monomer, M_w_ =32.9 kDa) agree well with the structure of MscS from *Escherichia coli* (1mxm.pdb). However, in contrast to *E. coli* MscS, T1 and T2 are predicted to contain an additional N-terminal transmembrane segment, TM0, resulting in a N_in_-C_in_-topology. TM3 shows the highest degree of sequence conservation between the three channels, a characteristic feature of MscS channels. T1 and T2 also lack the C-terminal beta strand that forms a seven stranded barrel in homoheptameric *E. coli* MscS. Furthermore, T2 contains an additional insertion of around 20 amino acids following the transmembrane region. 

### Characterization of purified T1 and T2

**Figure 2:**
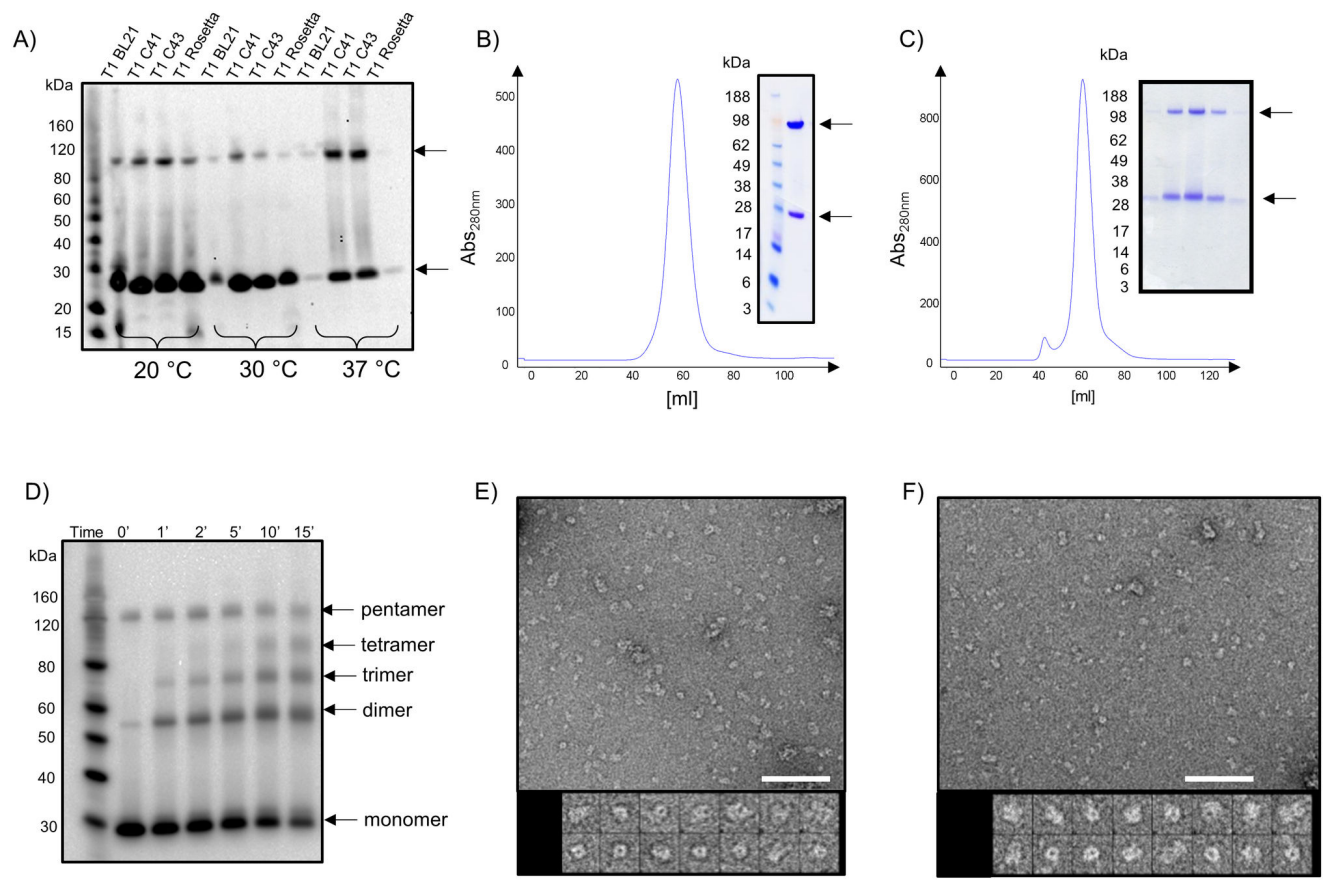
Expression, purification and characterization of the T1 and T2 channels from *Thermoplasma volcanium*. (A) Western blot of small scale expression screening of the T1 channel in four expressions hosts (BL21, C41, C43, Rosetta2) at three different expression temperatures (20 °C, 30 °C, 37 °C). Monomeric and oligomeric channel species are indicated with an arrow. (B, C) Both IMAC purified T1 (B) and T2 (C) channels elute as a single monodisperse peak from a preparative gel filtration column. Insets show SDS-PAGE from gel filtration fractions of both channels, confirming that the protein preparation is highly pure. (D) Crosslinking of the T2 channel in crude membranes suggests a pentameric architecture. (E, F) Negative stain EM of DDM solubilized T1 (E) and T2 (F) channels stained with 1% uranyl formate. Upon dilution the particles aggregated slightly. The scale bars represent 72 nm. The galleries show mostly tilted views, top views and some side views with dimensions of about 8 × 16 nm. The frame size of the boxed, magnified particles is 23 nm.

The position of the affinity tag, strain selection and induction temperature strongly influenced the amount and quality of over-expressed material. An N-terminally Histidine-tag was preferred compared to a C-terminal one (data not shown) and low induction temperature of 20 °C yielded substantial higher over-expressed material than higher temperatures (see Figure 2 A). When using the IMP optimized expression strain C41(DE3), more than 5 mg of purified channel protein could be obtained per 1 L of cell culture. Both archeal channels display a monodisperse elution profile on gel filtration (see Figure 2 B, C) and high stability against thermal unfolding (data not shown). This observation is also reflected in the migration behaviour on SDS-gels, where a monomeric and a higher oligomeric species are visible for both channels (see Figure 2 A-C). The distribution of oligomer versus monomer species on SDS-PAGE strongly depends on sample heating and SDS to protein ratio. Chemical crosslinking results in a ladder of five bands, where the highest band shows similar migration behaviour as the oligomeric species detected under non-crosslinking conditions (see Figure 2 D). These results support that T1 and T2 form pentamers, in contrast to the heptameric arrangement of *E. coli* MscS.

An oligomeric arrangement and homogeneity of the purified samples is further supported by electron microscopy. The reported small mechanosensitive heptameric channel have three domains with overall dimensions of 12 by 8 nm [38,39,48], which corresponds well with the particle dimensions observed with negative stain EM (see Figure 2 E, F). Contributions to the size from the stain and the detergent surrounding the transmembrane domains can make the particles appear larger. The channels were observed from all angles although with a predominance for tilted views (Figure 2 E, F). 

### Selection of T2 specific nanobodies

Despite extensive efforts of crystallization, neither of the archeal channels yielded diffracting crystals. We therefore generated nanobodies against the T2 channel for the use as crystallization chaperones that could potentially increase the chances of obtaining crystals. A llama was immunized with detergent solubilized T2 channel solution over a period of 6 weeks. A phage display library of nanobodies was created and T2 channel specific nanobodies were selected via phage display. 144 randomly chosen colonies were grown for expression of their nanobody as soluble protein. Crude periplasmic extracts were tested in an ELISA, and 102 extracts were shown to be specific towards the T2 channel (for more details see “Materials and Methods” section). Sequence analysis revealed 33 different nanobodies against the T2 channel belonging to 15 different sequence sub-families (see Figure 1). 23 nanobodies were recloned for large scale expression, purification and characterization.

### Characterization of nanobody binding to T2

**Figure 3:**
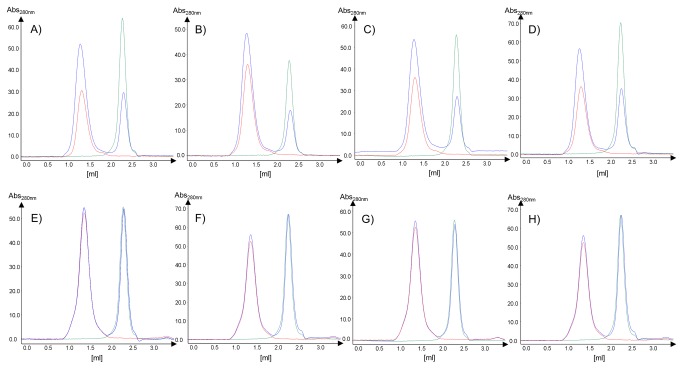
Binding of nanobodies to the T1 and T2 channels monitored *via* analytical gel filtration. Panels A-D represent elution profiles for the binding analysis of selected nanobodies against the T2 channel, while panels E-F display the same experiments but using the T1 channel as control (A, E: nanobody 15; B, F: nanobody 17; C, G: nanobody 19; D, H: nanobody 21). AGF profiles are shown for the nanobody only in green, for the T2/T1 channel only in red and the complex in blue.

All 23 nanobodies were expressed as soluble proteins in the periplasm of the *E. coli* strain WK6 and purified to homogeneity according to established methods. Typically 5 - 50 mg of purified nanobody was obtained from 1 liter of *E. coli* culture. All purified nanobodies were monomeric, highly stable and migrated according to their molecular mass on SDS-PAGE. To monitor binding of purified detergent solubilized T2 channel with various nanobodies *in vitro*, we first used an analytical gel filtration setup. The T2 channel was incubated with a 2-3 fold excess of nanobody for at least 60 min prior to analysis on the AGF column. Examples of the elution profiles for the complex, the T2 channel alone, and the nanobody alone are shown in Figure 3. Due to a size estimation of more than 200 kDa for the T2 channel detergent complex only a small shift to shorter retention times upon nanobody binding is detectable. However, the absorbance intensity for the T2 channel nanobody complex strongly increases compared to the uncomplexed T2 channel elution profile, while the peak height for the nanobody is strongly reduced. 22 out of 23 nanobodies formed a monodisperse complex as judged by the AGF analysis. As control experiment, none of the selected T2 nanobodies interacted with the homologous T1 channel (see Figure 3 E-H). 

**Figure 4:**
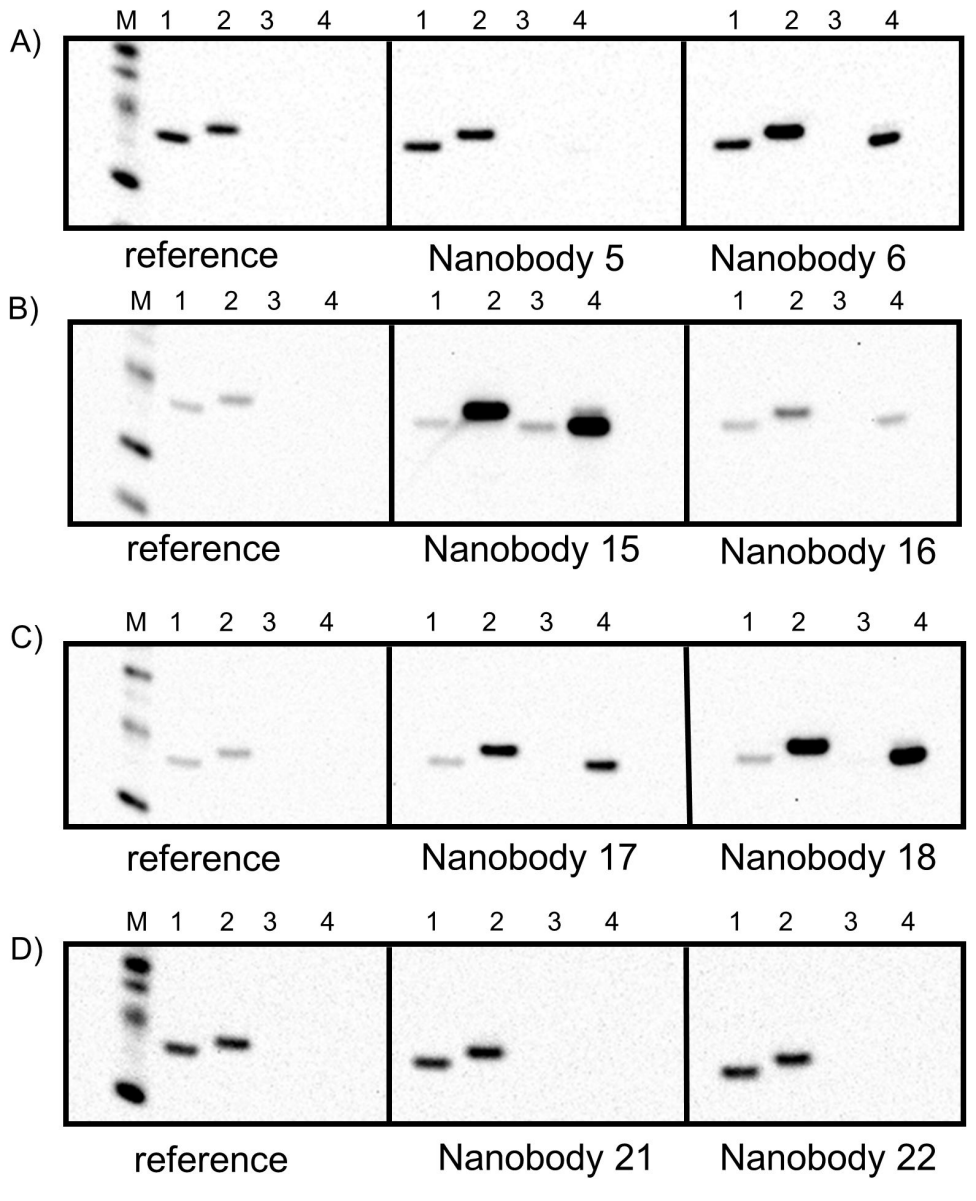
Western Blot analysis for affinity ranking of selected binders. Panels A-D show 4 Western blot examples using various nanobodies as stated. Each strip contains the same 4 samples: (1) His-tagged T1-channel (0.1 μg), (2) His-tagged T2-channel (0.1 μg), (3) tag free T2-channel at low concentration (0.01 μg), (4) tag free T2-channel at higher concentration (0.1 μg). The His-tagged molecular weight marker is shown on the reference strip. The western blot intensity ratios between lane two of the sample and reference strips allows a first ranking of binders according to their affinities.

To determine whether the selected binding proteins recognize a linear or conformational epitope on T2, Western blot analyses are typically performed. Here a denatured conformation of the antigen after SDS-PAGE and membrane transfer is assumed. Conformational epitope binders are expected to recognize the native state only, while nanobodies recognizing linear epitopes could be positive also in Western blot analysis. Instead of a standard Western blot analysis we developed a Western blot assay which not only allows us to differentiate between conformational and linear epitope binders, but also ranks the binders according to their expected affinity to the antigen. Each SDS-gel was loaded with three sets of four samples: His-tagged T1-channel (0.1 μg), His-tagged T2-channel (0.1 μg), tag free T2-channel at low concentration (0.01 μg), tag free T2-channel at higher concentration (0.1 μg). After the blocking step, the Western blot was divided in three strips (each containing one set of the four samples); one reference strip and two sample strips, thus allowing for the analysis of two different nanobodies per western blot with the negative control (see Figure 4)

**Figure 5:**
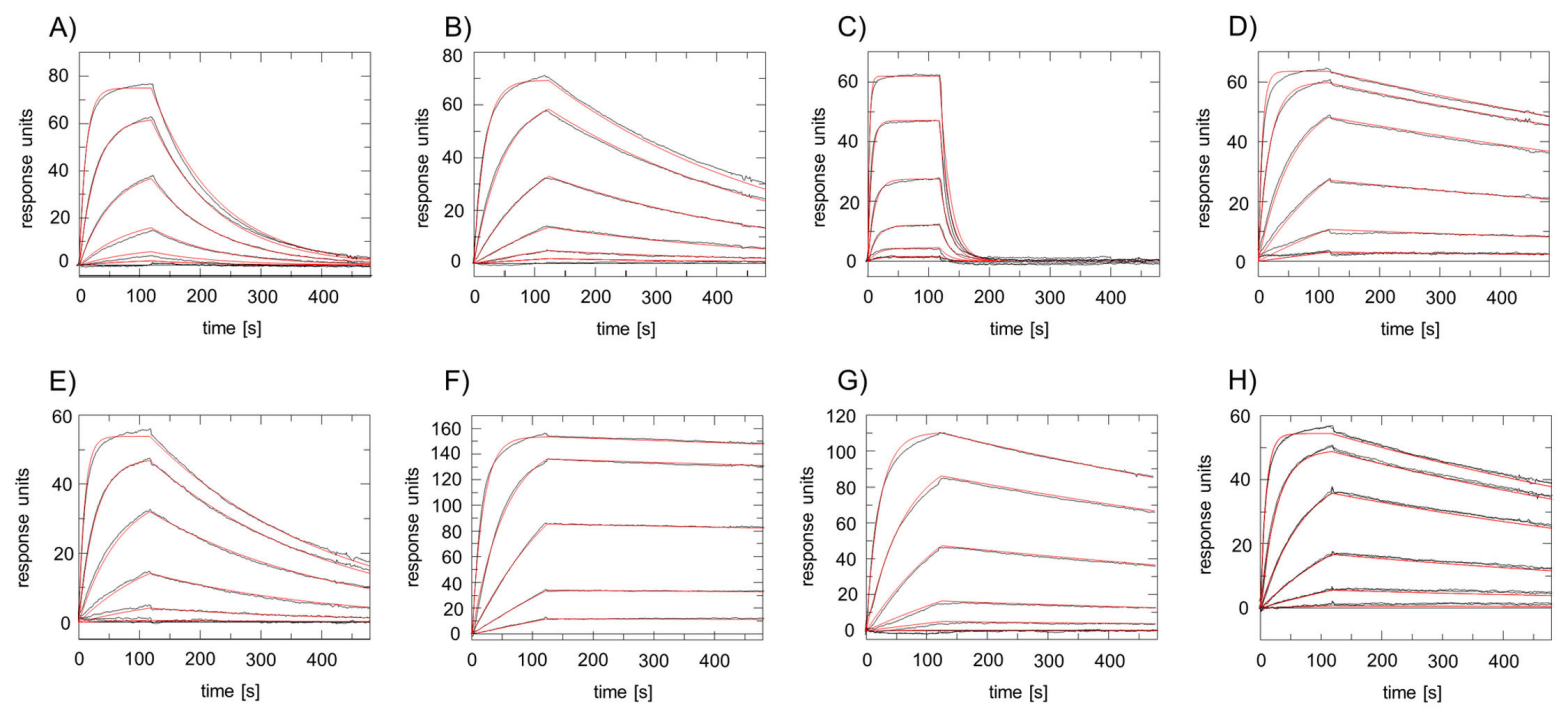
Surface plasmon resonance analyses of the T2 channel with various nanobodies. Different concentrations of purified nanobodies (0.35 - 86.1 nM) were injected across a surface coated with the T2 channel. Sensorgrams with blank buffer injections were subtracted. Panels A-H represent sensorgrams for nanobodies 3, 4, 7, 8, 10, 15, 18 and 23, respectively. Resulting on- and off-rates and dissociation constants are summarized in Table 1. Experimental curves are plotted in black; red lines denote the corresponding 1:1 interaction model fit to the experimental data.

**Figure 6:**
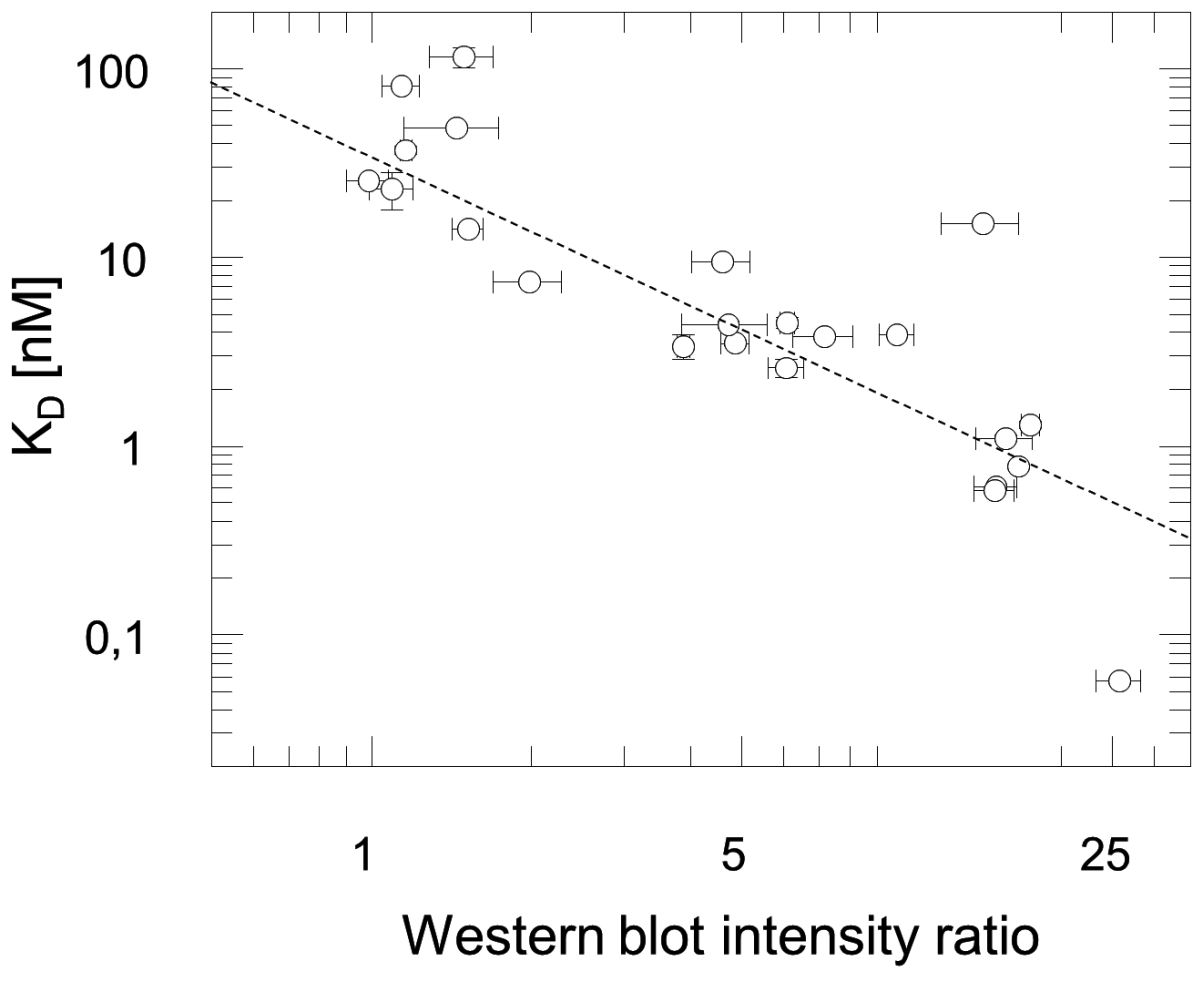
Correlation plot of affinity data. Ranking of affinities according to Western blot intensities agrees well with affinity data obtained from SPR analysis (χ=3.5, correlation coefficient r = -0.86).

After washing, all strips were incubated with a His-probe conjugated to horseradish peroxidase recognizing the Histidine tags of the bound nanobody and the His-tagged channels. The strips were finally combined before readout *via* chemiluminescence. Figure 4 shows examples of Western blots. Further analysis of this data not just allows the differentiation of conformational to linear epitope binders (e.g. nanobodies 5, 21 and 22 recognize conformational epitopes, while nanobodies 6, 15, 16, 17, 18 recognize linear epitopes), but also allows for a first ranking of binders with respect to their affinity (only valid for linear epitope binders). Here the band intensity ratio of lane 2 of the sample strips to the reference strip reveals valuable information. Tighter binding of the His tagged nanobody to the antigen leads to an increased band intensity due to increased binding of the HRP-conjugated His-Probe. Nanobody 15 shows the highest Western blot intensity ratio and is therefore predicted to be the tightest binder. To validate the ranking, we next determined the affinity values for each nanobody using surface plasmon resonance (see example sensorgrams in Figure 5). Kinetic data and resulting affinity values (*K*_D_) for all 23 binders are summarized in Table 1 and cover the range from 50 pM to 100 nM. Here, nanobody 15 was again identified as the binder with the highest affinity to the antigen. A correlation blot between the determined *K*_D_ values and the band intensity ratio of the Western blots confirmed that the binder ranking based on Western blot analysis agrees well with the SPR determined affinities (see Figure 6).

**Table 1:** Summary of T2/nanobody interactions.

Nb	CA-number	complex on AGF	pos. on WB	*k*_*a*_ x 10^5^ (M^-1^s^-1^)	*k*_*d*_ x 10^-3^ (s^-1^)	*K*_D_ (nM)	ratio of WB intensities
1	3379	+	-	19.8 ± 0.5	27.8 ± 0.8	14.2 ± 0.2	1.52 ± 0.10
2	3381	+	-	13.1 ± 0.2	104 ± 2	81.2 ± 0.4	1.14 ± 0.09
3	3382	+	+	9.6 ± 0.2	9.1 ± 0.7	9.5 ± 0.2	4.59 ± 0.57
4	3383	+/-	+	6.2 ± 0.4	2.1 ± 0.2	3.4 ± 0.5	3.87 ± 0.10
5	3386	+	-/+	1.5 ± 0.4	150 ± 10	115 ± 14	1.49 ± 0.20
6	3387	+	+	18.2 ± 0.3	8.1 ± 0.1	4.4 ± 0.2	4.70 ± 0.86
7	3388	+	-	30 ± 5	70 ± 7	23 ± 5	1.09 ± 0.10
8	3389	+	+	15.6 ± 0.4	0.89 ± 0.08	0.58 ± 0.01	14.96 ± 1.29
9	3390	+	-	31 ± 9	150 ± 2	48.3 ± 0.2	1.44 ± 0.29
10	3391	+	+	9.4 ± 0.3	3.6 ± 0.2	3.90 ± 0.08	9.80 ± 0.72
11	3393	+	+	1.0 ± 0.3	1.6 ± 0.3	15.06 ± 0.03	14.23 ± 2.36
12	3394	+	+	6.5 ± 0.3	2.5 ± 0.3	3.8 ± 0.1	7.15 ± 0.93
13	3395	+	+	14.6 ± 0.7	0.88 ± 0.013	0.61 ± 0.013	15.07 ± 1.39
14	3396	+	+	8.8 ± 0.1	2.3 ± 0.3	2.6 ± 0.3	6.07 ± 0.47
15	3397	+	+	19.64 ± 0.13	0.11 ± 0.02	0.057 ± 0.0018	25.74 ± 2.49
16	3398	+	+	7.5 ± 0.1	5.5 ± 0.2	7.4 ± 0.1	1.98 ± 0.29
17	3399	+	+	7.1 ± 0.6	3.15 ± 0.03	4.5 ± 0.3	6.09 ± 0.19
18	3401	+	+	4.8 ± 0.1	0.52 ± 0.02	1.10 ± 0.04	15.71 ± 1.93
19	3402	+	+	13.5 ± 0.8	1.5 ± 0.2	1.29 ± 0.06	17.48 ± 0.69
20	3403	+	+	10.3 ± 0.5	3.6 ± 0.3	3.5 ± 0.1	4.86 ± 0.29
21	3404	+	-	50 ± 4	171 ± 2	37 ± 2	1.16 ± 0.03
22	3406	+	-	2.4 ± 0.2	6.1 ± 0.2	25.41 ± 0.96	0.98 ± 0.09
23	3407	+	+	12.6 ± 0.2	0.95 ± 0.08	0.78 ± 0.01	16.60 ± 0.19

Results of analytical gel filtration (AGF), western blot analysis (WB) and kinetic rate constants and equilibrium dissociation constants as determined by SPR are shown. For experimental details see Material and Methods section.

### Large scale purification of T2/nanobody complexes and crystallization

**Figure 7:**
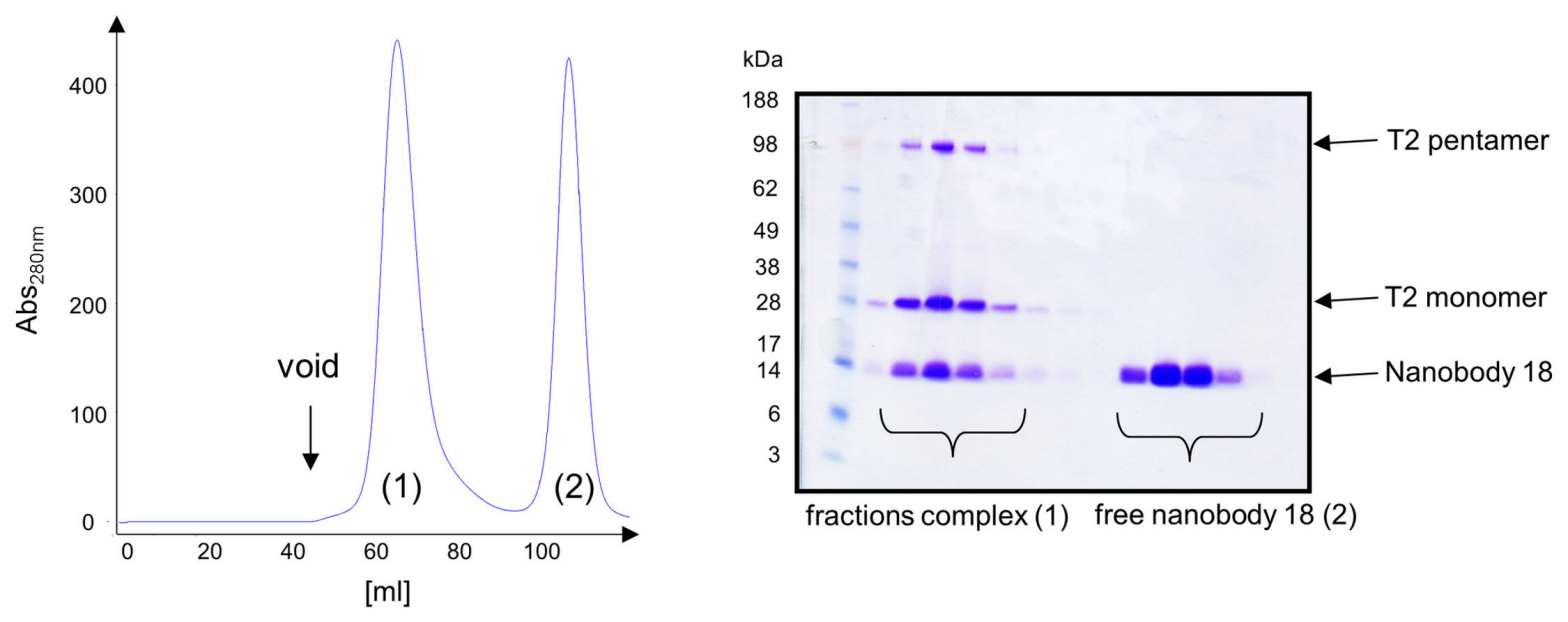
Preparative purification of T2/nanobody complexes. Purified T2 channel was incubated with an excess of nanobody (e.g. nanobody 18) prior to preparative gel filtration. Fractions containing channel protein and the nanobody were combined and used for crystallization studies. Preparative gel filtration profile (A) and SDS-PAGE analysis (B) of fractions is shown. Bands of corresponding proteins are labeled.

**Figure 8:**
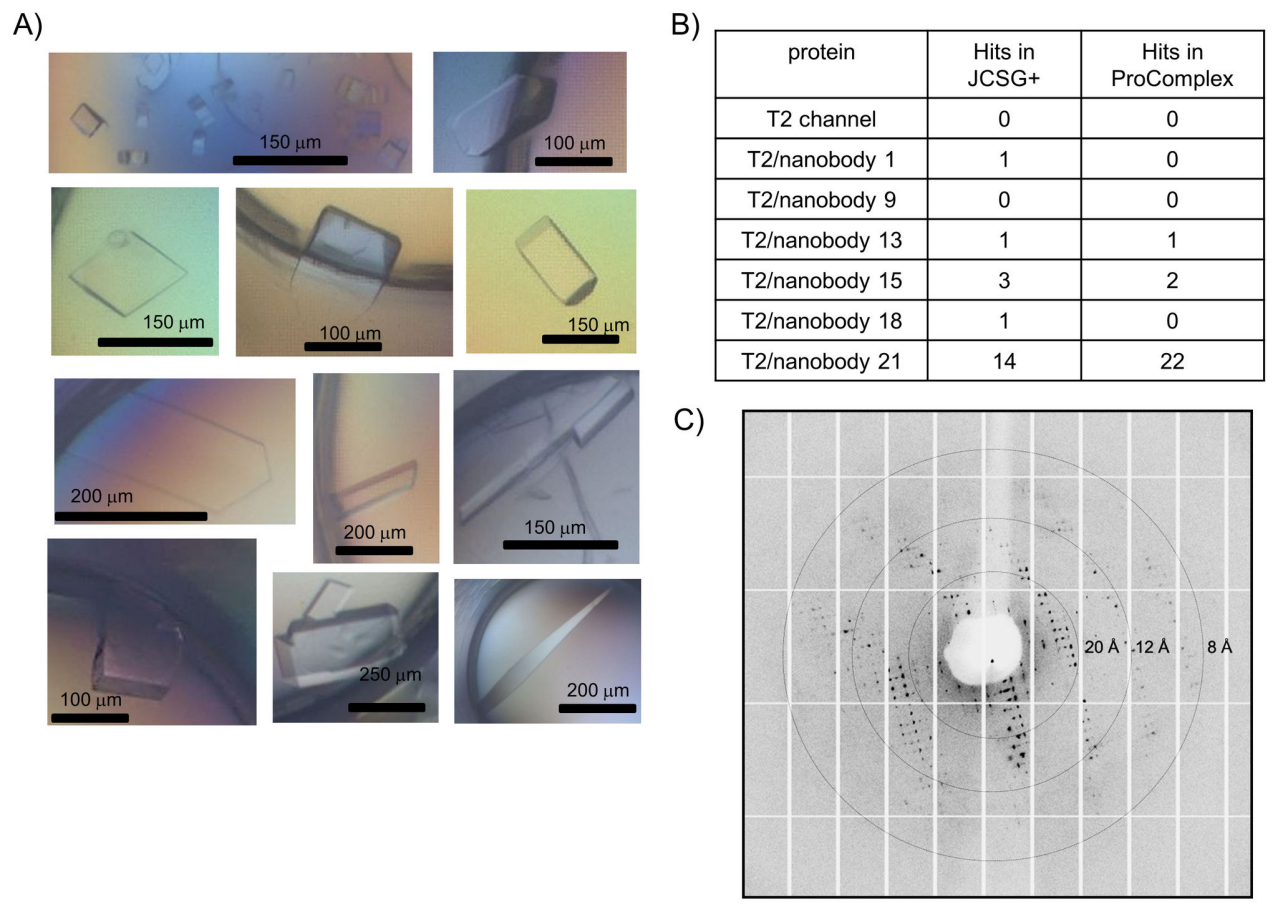
Crystallization of T2/nanobody complexes. (A) Examples of initial crystallization hits for different T2/nanobody complexes obtained in various conditions of the JCSG+ and ProComplex screens. The scale bar for size estimations of the crystal is shown. (B) Table with initial crystallization hits obtained for the different T2/nanobody complexes in the two commercially available crystallization screens JCSG+ and ProComplex. (D) Diffraction image from one of the crystals obtained from the initial crystal screening (T2/nanobody 21 complex) diffracting to 8 Å resolution.

From the 23 binders, we selected six nanobodies based on affinity and their binding mode (conformational binders preferred over linear epitope binders) for large scale preparation. To obtain various T2/nanobody complexes for crystallization trials, purified T2 channel was incubated with excess of nanobody prior to preparative gel filtration. Peak fractions containing stoichiometric amounts of both components were collected, concentrated and used for subsequent crystallization trials (see Figure 7 A). In contrast to crystallization trials with the T2 channel only, various T2/nanobody complexes gave rise to multiple crystallization hits within 3 weeks as illustrated in Figure 8. From the nanobodies tested, the conformational epitope binder, nanobody 21, had by far the highest impact on the crystallization behavior of the channel (see Figure 8 B). Crystals of the T2/nanobody complex diffracted X-rays to a resolution of 7 Å at best. A preliminary dataset was collected on a T2/nanobody21 crystal (see Figure 8 C) and could be processed to a max. resolution of 8.0 Å. This data set could be indexed to point group P222 with cell dimensions of a = 132.0 Å, b = 179.1 Å and c = 260.2 Å. Assuming a complex of five T2 subunits and five nanobodies (^~^ 250 kDa) either one or two complexes could fit in the asymmetric unit corresponding to a solvent content of 80 % or 60 % respectively (as determined from the Matthews coefficient).

## Discussion

### Recombinant over-expression of archeal mechanosensitive channels

The number of IMP structures solved to date has significantly increased in the last five years, due to a number of improvements in for example protein engineering, the development of new crystallization tools, microfocus X-ray diffraction, as well as NMR-methodology. Despite of these advances, obtaining novel crystal structures is still a tedious and very time-consuming process. The lack of structural data is a consequence of the many challenges IMP research is facing: toxicity to the over-production host, lipid requirements for correct folding and function, detergents destabilizing the IMPs and hampering the formation of well-ordered crystals. Strategies such as fusing green fluorescent protein (GFP) to IMPs have improved the screening process in identifying IMPs and constructs suitable for structural studies at an early stage in the process [49-53]. This gives preliminary information on the quality of the over-expressed IMPs and requires little material. However, the GFP pipeline in bacteria is limited to membrane proteins with a cytoplasmic C-terminus, since GFP can only fold correctly and become fluorescent when it is localized in the cytoplasm [54]. Indeed, both MS channels (T1, T2) from the archaea *Thermoplasma volcanium* described here have been screened for over-expression as C-terminal GFP fusion constructs previously [55], but were discarded due to insufficient expression levels. However, following our recently described high-throughput approach for identification of IMP targets for structural studies [47], which is independent of fluorescent fusion partners, we readily identified both channels as prime targets for structural studies. High expression levels (> 5mg/L of culture) and homogenous channel preparations after purification were achieved with N-terminally tagged constructs only, indicating that C-terminal tags or fusion partners might interfere with the correct folding and oligomeric assembly of the channel. Therefore, screening tools allowing for variable tag positions will identify more candidates suitable for structural studies as previously demonstrated [47].

### Characterization of nanobodies

The co-crystallization of binding proteins with IMPs is an emerging technique, which has led to structure determination of a diverse set of membrane proteins [11]. The formation of an IMP/binding protein complex can not only enhance the solubility of IMPs and stabilize certain conformations, but also increases the hydrophilic protein surface for crystal contact formation. Typically there are only very few hydrophilic residues available in IMPs for crystal contacts due to the presence of detergents covering the surface of the transmembrane region. Various scaffold proteins have been used to enhance crystallization propensities of IMPs. The advantage of nanobodies as used here is their exceptionally high affinity, high stability and ability to bind to regions inaccessible for conventional antibodies. Furthermore they can be produced in high amounts at low cost in the bacterial host *E. coli* [56]. This approach was for example crucial for stabilizing the active conformation and obtaining well-diffracting crystals of β2-AR and in complex with a G-protein [28,29]. In general, conformational binders are favored compared to linear epitope binders since they are expected to reduce conformational flexibility [57]. Usually Western blot analysis is employed to distinguish between the different binding modes. Nevertheless it should be noted, that IMPs often retain a significant amount of secondary and tertiary structure upon SDS treatment [58,59]. Furthermore it cannot be excluded that certain proteins/domains refold upon Western blot transfer in aqueous solution and contain a significant fraction of secondary and tertiary structure. Thus potential conformational epitope binders may be misleadingly classified as linear epitope binders. Out of 23 binders tested against the target protein T2, six unique binders were negative in our Western blot analysis but formed complexes with the native antigen as judged by analytical gel filtration. The initial characterization of binders using automated analytical gel filtration and the modified Western blot protocol allowed us to identify the most promising binders for crystallization studies in a fast manner. The affinity ranking of binders was confirmed with affinity data derived from SPR or ITC measurements (ITC data not shown). Therefore we conclude that a combination of methods such as automated analytical gel filtration and Western blot analysis is sufficient for screening binding proteins in the first place, although SPR and ITC provide deeper insights into affinity and potential binding mode of the binders. However these tools require significantly higher amounts of material and due to the presence of detergents the analysis of these data is often more challenging and time consuming. 

### Nanobodies as crystallization chaperones

Several examples of chaperone mediated crystallization of IMPs have been reported in the literature, with the first successful being the 2.7 Å resolution structure of *Pseudomonas denitrificans* cytochrome c oxidase (COX) catalytic subunit [60]. Here, a selected antibody fragment mediated most of the crystal contacts and allowed its structure determination. Further successful examples mainly made use of antibody fragments derived from hybridoma technology [61,62] and recent examples used selected binding proteins based on proteins scaffolds such as DARPins [63] or fibronectin [64]. Camelid nanobodies become increasingly popular due to the recent successes in the field of GPCR structural biology [28,29]. It should be noted, that crystallization chaperones often help to improve resolution of IMP structures due to restriction of conformational flexibility and allocation of additional crystal contacts. But in the reported cases, initial crystals diffracting to lower resolution were most often already obtained for the IMPs only. This is rather different in our case for both mechanosensitive channels, where no initial crystal hits could be identified despite extensive screening. Similar results for these channels have recently been reported [40]. However, the presence of a nanobody had dramatic effects on the crystallization propensity resulting in a number of crystallization leads representing various crystal forms. Crystallization conditions covered the pH range from 4 - 7 in combination with various PEGs as precipitants (PEG400 – PEG8000). Therefore it is tempting to speculate that different states of the channels might be captured e.g. in the presence of different nanobodies or crystallization conditions. Further crystal optimization will be necessary to get crystals diffracting to higher resolution and subsequent structure determination. Here we have shown that nanobodies can dramatically widen the crystallization space of the highly challenging class of integral membrane proteins and we are convinced that this tool will have a dramatic effect for IMP structure determination in the future. 

## Supporting Information

Figure S1 **Sequence alignment of all 23 selected T2 specific binders.** Corresponding CA-numbering for each nanobody can be found in Table 1.
(PDF)
