# Supplementary material for: Nanobody Mediated Crystallization of an Archeal Mechanosensitive Channel
Source: PLoS One. 2013 Oct 21;8(10):e77984. doi: 10.1371/journal.pone.0077984 (PMC3804602; doi:10.1371/journal.pone.0077984)
Supplement: Figure S1 — Sequence alignment of all 23 selected T2 specific binders. Corresponding CA-numbering for each nanobody can be found in Table 1. (PDF) [file pone.0077984.s001.pdf]

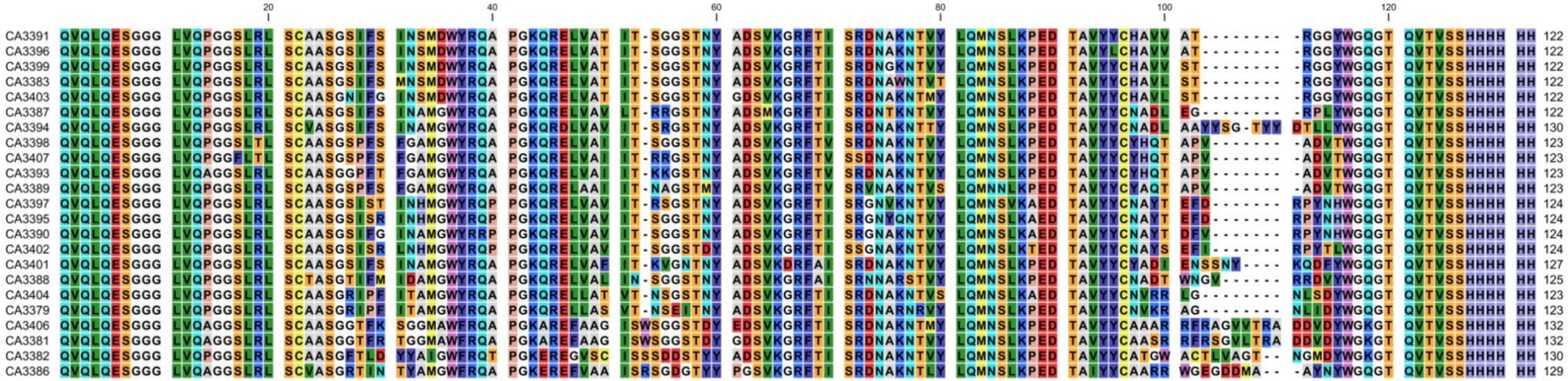

Supplementary Fig. S1. Sequence alignment of all 23 selected T2 specific binders. Corresponding CA-numbering for each nanobody can be found in Table 1.
